# Neonatal jaundice: magnitude of the problem in Cairo University's neonatal intensive Care unit as a referral center

**DOI:** 10.4314/ahs.v23i1.70

**Published:** 2023-03

**Authors:** Emad Emil Ghobrial, Hashem Mohamed Al Sayed, Abd Elmeneim Mohamed Saher, Badr El-Din Reem Mahmoud

**Affiliations:** 1 Department of Pediatrics, Faculty of Medicine, Cairo University, Cairo; 2 Department of Pediatrics, Ministry of Health, Cairo

**Keywords:** Indirect hyperbilirubinemia, phototherapy, exchange transfusion

## Abstract

**Background:**

Neonatal jaundice is one of the most common physiologic problems requiring medical attention in newborns. It is benign in most cases; however, high levels of bilirubin are neurotoxic and can lead to serious brain damage.

**Objectives:**

This study aimed at assessment of magnitude of neonatal jaundice in cases of neonatal hyperbilirubinemia admitted into neonatal intensive care unit (NICU), Cairo University Pediatric Hospital and to detect possible etiologies, management and outcome.

**Methods:**

The present work is a retrospective study, included 789 neonates suffered from hyperbilirubinemia over a two-year period.

**Results:**

Intensive phototherapy and exchange transfusion were used together in 6 cases. Two hundreds and twenty-two cases (28.1%) had exchange transfusion once, 44 cases had it twice, 6 cases had it 3 times and one case had it 4 times. Number of exchange transfusion significantly affects mortality among cases (P= 0.02).

**Conclusion:**

Neonatal hyperbilirubinemia is an existing problem in our NICU. Intensive phototherapy is an excellent substitute for exchange transfusion. Respiratory distress and sepsis are significantly higher among dead cases. Screening for risk factors is needed to avoid critical hyperbilirubenemia.

## Introduction

Jaundice is one of the most common physiologic problems requiring medical attention in the newborn. All infants, term or preterm, healthy or ill, undergo changes in bilirubin metabolism after birth. These normal transitional changes may lead to physiologic jaundice 1. Epidemiologic studies show that about 60% of term and 80% of preterm babies develop jaundice in the first week of life 2

Neonatal jaundice can be best understood as a balance between the production and elimination of bilirubin, with a multitude of factors and conditions affecting each of these processes 3.

Neonatal jaundice is benign in most cases; however, high levels of bilirubin are neurotoxic and can lead to serious damage to the brain 4.

Unconjugated bilirubin has a neurotoxic potential because of the ability to cross the blood-brain barrier and can cause kernicterus (chronic bilirubin encephalopathy)^5^. No clear-cut level of bilirubin above which encephalopathy is assured and below which neurologic safety is assured has been determined 6.

Although most of newborns develop some degree of jaundice, bilirubin levels high enough to put a newborn at risk of bilirubin encephalopathy and kernicterus are rare but still occur in Egypt 7.

Iskander et al in 2012 8 reported that the causes of indirect hyperbilirubinemia were ABO incompatibility (25.4%), Rh incompatibility (8.5%) with most mothers had blood group A -ve (3.6%) then O -ve and B -ve (each was 1.7%), sepsis (9.2%), dehydration (13.8%), polycythemia (4.6%), bruising (7.7%), other hemolytic disorders (5.4%), breast milk (1.5%) and undetermined (40%).

Regardless to the cause of indirect hyperbilirubinemia, the goal of therapy is to prevent neurotoxicity 9. Phototherapy and, if unsuccessful, exchange transfusion remains the primary treatment modalities to keep maximal total serum bilirubin (TSB) below pathologic levels^4^. High-intensity phototherapy significantly reduces TSB and decreases the need for exchange transfusion 10.

When serum bilirubin is rising rapidly or approaching exchange transfusion level, intensive phototherapy must be instituted at maximal spectral power 11.

The aim of current study was to assess the magnitude of neonatal jaundice and detect possible etiologies, management and outcome.

## Patients and methods

This retrospective study included all neonates suffered from neonatal hyperbilirubinemia admitted to neonatal intensive care unit (NICU) of Cairo University Pediatric Hospital (CUPH) over a two-year period from January 2010 to December 2011.

Neonates with life-threatening congenital anomalies and neonates presented with signs or symptoms suggestive of central nervous system abnormality due to other causes (e.g. hypoxic ischemic encephalopathy) were excluded from the study, because these neonates may have neurological symptoms and signs which may be mistaken with those due to kernicterus.

When an infant's serum bilirubin is rising rapidly, intensive phototherapy must be instituted at maximal spectral power [11]. American Academy of Pediatrics [6] recommends performing exchange transfusion for full-term healthy newborns at least 4 days of age if their TSB level is 25 mg/dl or more and does not decrease sufficiently with phototherapy alone.

Data of each patient were recorded from their files including;

a) **History taking;** date of birth, sex, gestational age, mode of delivery, history of maternal illness and/or drugs, previous siblings with jaundice, symptoms suggestive of kernicterus (in the form of poor suckling, lethargy, hypotonia, hypertonia, arched back and seizures), time of onset of jaundice and any associated symptoms (e.g. respiratory distress).

b) **Clinical examination;** anthropometric parameters, neonatal reflexes, extent of jaundice, pallor and neurological examination, in addition to chest, cardiac and abdominal examination.

c) **Laboratory investigations;** complete blood count, reticulocytic count, maternal and infant blood group (ABO and Rh typing) and blood culture for patients suspected to have sepsis.

d) **Therapy** in the form of phototherapy and its duration with or without exchange transfusion.

e) **Outcome** whether died or living, and the living cases whether normal or discharged with neurological sequelae.

## Statistical analysis

Statistical Package for Social Science (SPSS v20) was used after transforming the data from Excel 2013 sheet. Categorical variables were presented by number and percent. They were compared using Chi-square test or Fischer's exact test when appropriate. Continuous variables were presented by mean and standard deviation or median and range. They were compared by student's t-test if parametric data and using Mann Whitney U test if non parametric data. In all tests, P value was considered significant if less than 0.05.

## Results

**Figure 1:**
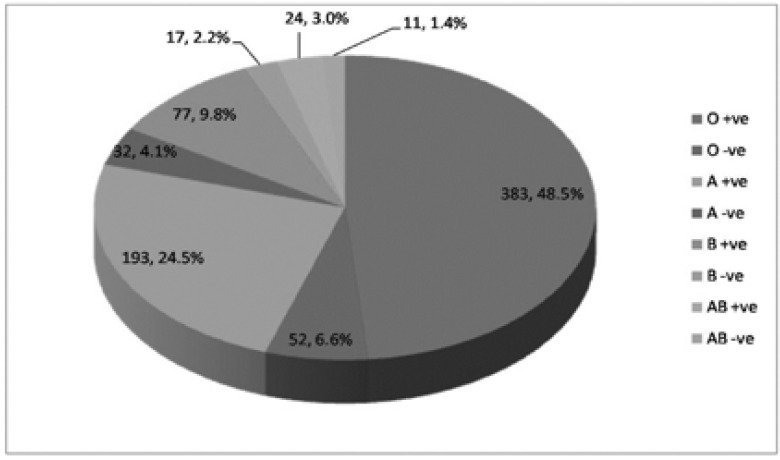
Blood group and Rh typing of the mothers.

**Figure 2:**
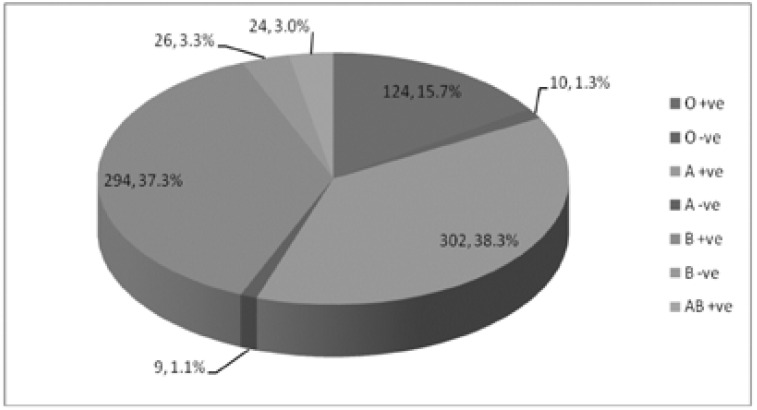
Blood group and Rh typing of the neonates.

In this study, we studied 789 neonates with hyperbilirubinemia, with demographic and medical data as shown in table (1). Table (2) shows clinical data of the patients. Figure (1) shows the blood group and Rh group of the mothers and figure (2) shows the blood group and Rh group of the neonates. Three hundred and thirty-five cases (42.5%) had ABO incompatibility, sixty-four cases (8.1%) had Rh incompatibility and twenty-seven cases (3.4%) had combined ABO and Rh incompatibility. Table (3) shows laboratory investigations of the patients.

**Table 1:** Demographic and medical data of the cases

Parameter		Frequency (n=789)
**Sex**	Male	440 (55.8%)
	Female	349 (44.2%)
Birth Weight (kg) (Mean ± SD)		2.79 ± 0.68
**Mode of Delivery**	CS	294 (37.3%)
	Vaginal	495 (62.7%)
**Gestational Age** **(weeks)**	Mean ± SD	37.59 ± 1.88
	Preterm (<37)	133 (16.9%)
	Full Term (37–41)	656 (83.1%)
**Medical history**	Maternal illness and/or drugs	18%
	Maternal obstetric complications	7.7%
	Siblings with jaundice	5.7%
	Difficult labour	1.6%

SD: Standard deviation

CS: Cesarean section

**Table 2:** Clinical data of the studied cases

Parameter		Mean ± SD
Day of Onset of Jaundice		2.78 ± 2.34
Total Bilirubin Level at Presentation(mg/dL)		25.15 ± 7.22
Maximum Total Bilirubin Level (mg/dL)	Value	25.76 ± 7.13
	Day of Detection	6.18 ± 3.76
**Signs of kernicterus**		
Poor suckling		49 (6.2%)
Lethargy		34 (4.3%)
Hypotonia		6 (0.8%)
Arched back		28 (3.5%)
Hypertonia		27 (3.4%)
Seizures		33 (4.2%)
**Clinical data**		
Respiratory distress		25 (3.2%)
Sepsis		55 (7%)
Pallor		91 (11.5%)
Cephalhematoma		4 (0.5%)
Tachycardia		8 (1%)
Abdominal distension		21 (2.7%)

**Table 3:** Laboratory investigations

Parameter	Mean± SD
Hemoglobin (g/dL)	13.3 ± 2.94
Total leucocytic count (10^3^/mm^3^)	10.6 ± 4.63
Platelets (10^3^/mm^3^)	274.5 ± 115.39
Reticulocytic count (%)	6.5 ± 9.12
ALT (IU/L)	24.9 ± 41.1
AST (IU/L)	62.4 ± 73.35
Albumin (g/dl)	3.1 ± 0.52
Sodium (mEq/L)	137.4 ± 1.54
Potassium (mEq/L)	6.6 ± 1.99
Positive blood culture	11 cases (1.4%)
CONS	5 cases (0.6%)
Enterococci	1 case (0.1%)
Klebsiella	1 case (0.1%)
MRSA	1 case (0.1%)
Pseudomonas	1 case (0.1%)
Staphylococcus aureus	1 case (0.1%)
Staphylococcus epidermidis	1 case (0.1%)

SD: Standard deviation

ALT: Alanine transaminase

AST: Aspartate transaminase

CONS: Coagulase Negative Staphylococcus aureus

MRSA: Methicillin Resistant Staphylococcus aureus

Table (4) shows data of phototherapy (conventional and Bilisphere), and exchange transfusion. Bilisphere and exchange transfusion were used together in 6 cases; all of them used exchange once, all of them were with maximum bilirubin level above 30 mg/dL except one preterm case (28 weeks gestational age) with maximum level 16.2 mg/dL.

**Table 4:** Phototherapy (conventional and Bilisphere) and Exchange Transfusion

Parameter		Mean ± SD
**Phototherapy** **(Days)**	Single	1.1 ± 1.32
	Double	1.8 ± 1.63
	Triple	0.4 ± 0.83
**Exchange** **Transfusion**	Times	1.2 ± 0.49
	Onset of the First (Day of Life)	5.29 ± 3.115
	Exchange once	222 (28.1%), 6 died
	Exchange twice	44 (5.6%), none died
	Exchange 3 times	6 (0.8%), 1 died
	Exchange 4 times	1 (0.1%), died
**Bilisphere**	Days (Mean ± SD)	0.2 ± 0.46
	For 1 Day	127 (16.1%)
	For 2 Days	15 (1.9%)
	For 3 Days	1 (0.1%)

Table (5) shows data of outcome of the patients (including duration of hospital stay). The outcome of the patients was as follows; 746 (94.6%) were normal on discharge, 20 (2.5 %) died and 23 were discharged with neurological sequelae [6 (0.8%) with poor feeding, 5 (0.6%) with tone abnormalities and 12 (1.5%) with controlled seizures].

**Table 5:** Outcome of the patients

Parameter		Frequency (n=789)
Duration of hospital stay (days) Mean ± SD		4.96 ± 4.60
Normal at discharge		746 (94.6%)
Neurological sequelae	Poor feeding	6 (0.8%)
	Tone abnormalities	5 (0.6%)
	Seizures (controlled)	12 (1.5%)
Died		20 (2.5%)

There were two cases used Bilisphere and died. One case used bilisphere and was discharged with poor feeding. Average hospital stays of cases used Bilisphere (143 cases) was 3.81 days and average hospital stay of the other cases (646 cases) was 5.21 days.

Table (6) shows the comparison of the outcome of cases in relation to number of times of exchange transfusion. The number of exchange transfusion significantly affects the mortality among cases with P value 0.02.

**Table 6:** Comparison of the outcome of cases in relation to number of times of exchange transfusion

Number of times of exchange transfusion	Outcome		p-value
	Alive (n=265)	Dead (n=8)	
1 (222 cases)	216 (97.3%)	6 (2.7%)	0.02
2 (44 cases)	44 (100%)	0 (0.0%)	
3 (6 cases)	5 (83.3%)	1 (16.7%)	
4 (1 case)	0 (0.0%)	1 (100%)	

The number of patients who had respiratory distress and number of patients who had sepsis were significantly higher among dead cases with P value < 0.001. The number of patients who had ABO incompatibility and combined ABO and Rh incompatibilities did not significantly affect mortality among cases with P value 0.11 and 0.39, respectively, while the number of patients who had Rh incompatibility were significantly higher among dead cases with P value 0.005.

The number of days of Bilisphere use did not significantly affect the mortality among cases with P value 0.17.

## Discussion

Early management with phototherapy alone or with exchange transfusion in indicated cases significantly decline the total serum bilirubin levels on follow up of cases and improve the outcome 7.

In our study, almost all patients received phototherapy (99.5%). One hundred and forty-three patients (18.1%) were managed via extensive phototherapy (Bilisphere). Two hundred and seventy-three patients (34.6%) had exchange transfusion. The mean number of exchange transfusion sessions was 1.2 ± 0.49 sessions.

The high incidence of exchange transfusion is due to the late use of Bilisphere in our NICU of CUPH; Bilisphere was brought to the NICU of on September 2011.

Bilisphere caused marked reduction in bilirubin level in a short period and decreased the period of hospital stay. Therefore, it decreased the need for exchange transfusion and helped the rapid discharge of neonates. Mean duration of hospital stay was 4.96 ± 4.6 days.

Seoud et al., 2007 10 found that exchange transfusion was done in 22.5% of the jaundiced patients.

On the contrary, the percentage was lower in the study conducted by Owa and Ogunlesi in 2009 11 (only 5.3% of the neonates received exchange transfusion), but with similar mean number of exchange transfusion sessions (1.2 ± 0.43 sessions).

In the present study, there were six cases received both Bilisphere and exchange transfusion, all of them used exchange once.

American Academy of Pediatrics (AAP), (2004) 5 recommends performing exchange transfusion for full-term healthy newborns at least 4 days of age if their TSB level is 25 mg/dl or more and does not decrease sufficiently with phototherapy alone.

In the current study, the mean duration of single phototherapy was 1.1 ± 1.32 days, of double phototherapy was 1.8 ± 1.63 days and of triple phototherapy was 0.4 ± 0.83 days, while the mean duration of Bilisphere was 0.2 ± 0.46 days.

In the study done by Abd-Ellatif et al., 2012 12, the mean duration of intensive phototherapy use was 14 hours.

In the current study, the mean gestational age was 37.59 ± 1.88 weeks and 83.1% of cases were full term.

In a study by Davutoglu et al., 2010 13 on 79 neonates had exchange transfusion during a five-year period from 2003 to 2008, the mean gestational age was 37.0 ± 2.1 weeks, and only 15.2% were below 38 weeks of gestation.

Henny-Harry and Trotman, 2012 14 stated that the mean gestational age was 36.2 ± 3.1 weeks, with 57.6% of caes were full term gestation. The AAP in 2004 5 considered gestational age 37-38 weeks a minor risk factor for development of severe hyperbilirubinemia and the gestational age 35-36 weeks a major risk factor.

In our study, there was male predominance, with 440 (55.8%) males and 339 (44.2%) females. The percentage was very near to the study by Henny-Harry and Trotman, 2012 14; 61% were males and 39% were females. In Gamaleldin et al., 2011 15, 54.2% were males and 45.8% were females. In another study done by Heydarian and Majdi in 2010 16 on 118 neonates weighting 2 kg and more, who had exchange transfusion 63.6% were males and 36.4% were females.

The AAP in 2004 5 considered male gender a minor risk factor for development of severe hyperbilirubinemia.

In the current study, 62.7% of cases were delivered vaginally and 37.3% were delivered by cesarean section.

Near to our results, Gamaleldin et al., 2011 15 found that there were 73.3% of cases were delivered vaginally. Iskander et al., 2012 17, found that 72.3% of cases were vaginally delivered and concluded that vaginally delivered babies are likely to be discharged early from hospital and this may cause delayed diagnosis of jaundice and increased risk of kernicterus.

In the present study, there were 45 (5.7%) cases had siblings with neonatal jaundice. This is close to what Henny-Harry and Trotman, 2012 14 found that 4% of the cases had siblings treated for hyperbilirubinemia.

The AAP in 2004 5 considered presence of previous siblings with jaundice a minor risk factor for hyperbilirubinemia and presence of siblings treated with phototherapy a major risk factor.

In the current study, the total bilirubin level on admission was 25.15 ± 7.22 mg/dL and the maximum total bilirubin level was 25.76 ± 7.13 mg/dL.

Similar to our results, Iskander et al., 2012 17 found that total bilirubin on admission was 25.76 ± 4.39 mg/dL. Davutoglu et al., 2010 13 reported higher level of bilirubin; the mean peak total bilirubin level was 28.1±6.4 mg/dL.

The mean age of onset of jaundice in our study was 2.78 ± 2.34 days. On the contrary, Iskander et al., 2012 17 found that the mean age of onset of jaundice was 4.1 ± 3.4 days. Similar to this result, Davutoglu et al., 2010 13, found that the mean age of onset of jaundice was 4.9 ± 2.2 days.

The mean age of maximum serum bilirubin level in our study was 6.18 ± 3.76 days. Henny-Harry and Trotman, 2012 14 reported a younger age at which maximum bilirubin level was reached (4.7 ± 2.1 days).

In the present study, the common presenting symptoms of newborns with kernicterus were as follows; 6.2% of cases had poor suckling, 4.3% had lethargy, 0.8% had hypotonia, 3.5% had arched back, 3.4% had hypertonia and 4.2% had seizures.

Weng and Chiu, 2009 18 found that the main symptoms noticed were tachypnea (6%), apnea (2.4%), fever (1.2%), irritability (2.4%), lethargy (4.8%), poor feeding (19.3%) and seizures (1.2%).

Hashem et al in 2009 19 at NICU of CUPH reported higher percentages of symptoms in newborns with kernicterus; poor feeding (94.4%), irritability (83.3%), abnormal movements and seizures (50%), opisthotonus (33.3%), hypotonia (27.7%) and apnea (22.2%).

In the current study, 55.1% of mothers had blood group O and 14.3% Rh negative mothers. The majority of neonates in our study had group B (40.6%) and A (39.4%) then O (17%).

The percentage of maternal blood group O was also the highest in the study done by Henny-Harry and Trotman, 2012 14 (66%) and 12% were Rh negative mothers.

In the current study, the cause of indirect hyperbilirubinemia was ABO incompatibility in 335 (42.5%) cases, Rh incompatibility in 64 (8.1%) cases, combined ABO and Rh incompatibility in 27 (3.4%) cases, sepsis in 55 (7%) cases and cephalhematoma in 4 (0.5%) cases. The rest of the cases (38.5%) were with an unidentified cause.

Similarly, Iskander et al., 2012 17 reported undetermined cause in 40% of cases. Higher percentage of the unknown cause of neonatal jaundice was reported by Gamaleldin et al in 2011 (49.8%) 15.

On the contrary, Davutoglu et al., 2010 13 reported that among causes of indirect hyperbilirubinemia, 13.9% were unknown.

The high prevalence of ABO and Rh incompatibility in the studies done in NICU of CUPH can be attributed to lack of proper antenatal care and lack of awareness of general population.

Septicemia was an important cause of hyperbilirubinemia among our patients, being found in 7% of cases. Similarly, Seoud et al., 2007 10 reported septicemia in 7.7%. The prevalence of sepsis in different studies in Iran, Turkey and Canada were 15.7%, 7.5% and 3.9% respectively 20,21, 22.

Lack of hygiene during and after delivery, poor cord care and unhygienic newborn care practices may be the major factors in acquisition of neonatal infections and sepsis in both hospital and community settings 23. Lack of proper antenatal care by mothers and absence of being screened for infections and other risk factors may affect their babies^24^.

The outcome of our cases study showed that 20 patients died (2.5%) and 769 patients (97.5%) were alive; among them six (0.8%) patients were discharged with poor feeding, five (0.6%) had tone abnormality and twelve (1.5%) had seizures, controlled on anticonvulsant therapy.

Henny-Harry and Trotman, 2012 14 found that there were two deaths, but these were related to extreme prematurity and only one patient was discharged with bilirubin encephalopathy.

In the present study, the number of patients who had respiratory distress and number of patients who had sepsis was significantly higher among dead cases (P value < 0.001).

The number of patients had ABO incompatibility and combined ABO and Rh incompatibilities did not significantly affect the outcome of the cases, while the number of patients had Rh incompatibility had a significant correlation with the outcome of cases of neonatal jaundice. The number of days of Bilisphere use did not significantly affect the mortality. This may be due to the small number of cases used Bilisphere in relation to the total number.

In the present study, two hundreds and twenty-two cases (28.1%) had exchange transfusion once (6 of them died), 44 cases (5.6%) had it twice (none of them died), six cases (0.8%) had it three times (one of them died) and one case (0.1%) had it four times (died). The number of exchange transfusion significantly affects the mortality among cases with P value 0.02.

Helal et al. (2018) 25 stated that acute bilirubin encephalopathy (ABE) is still a major problem in Egypt. They reported that overall mortality was 9.9% and concluded that admission total serum bilirubin and maternal illiteracy are good predictors of bilirubin encephalopathy at admission ad discharge.

Iskander et al. (2012)^17^ stated that in Egypt, several other factors predispose to severe hyperbilirubinemia and kernicterus. These include inappropriate maternal knowledge about the possible risks of severe neonatal jaundice, delay in seeking medical advice, home therapy for jaundice using neon lamps that do not provide the required wavelength, and difficulty in finding adequate medical care especially in remote areas when required.

Iskander et al. (2014) 26 stated that in Egypt, severe hyperbilirubinemia and bilirubin encephalopathy are still being reported in numbers that cannot be ignored.

In Egypt, screening for risk factors of severe hyperbilirubinemia is not routinely done for newborn before discharge from the delivery hospital. The goal of screening for neonatal jaundice is to promote early identification and prompt intervention to avoid severe or critical hyperbilirubinemia that causes kernicterus 27.

Basheer et al.^28^ explained lack of screening by that since health policies worldwide have changed favoring shorter hospital stays and early discharge for mothers and their newborns after delivery; kernicterus, which was thought to have almost completely disappeared, remerged and is of greater concern for neonatologists and pediatricians. The major limitation of this study was the lack of follow up of patients after discharge.

## Conclusion

The present study proved that neonatal indirect hyperbilirubinemia is an existing problem in our NICU related to many etiological factors. Respiratory distress and neonatal sepsis are significantly higher among dead cases. We also concluded that intensive phototherapy is a good substitute for blood exchange. Screening for risk factors for neonatal jaundice is needed to avoid critical hyperbilirubenemia.
